# Incorporating patient experience into drug development for ulcerative colitis: development of the Urgency Numeric Rating Scale, a patient-reported outcome measure to assess bowel urgency in adults

**DOI:** 10.1186/s41687-022-00439-w

**Published:** 2022-04-01

**Authors:** Marla C. Dubinsky, Peter M. Irving, Remo Panaccione, April N. Naegeli, Alison Potts-Bleakman, Vipin Arora, Mingyang Shan, Simon Travis

**Affiliations:** 1 Department of Pediatrics, Susan and Leonard Feinstein IBD Center, Icahn School of Medicine, Mount Sinai, 17 East 102nd Street, 5th Floor East, New York, NY 10029 USA; 2 Department of Gastroenterology, Guy’s and St Thomas’ NHS Foundation Trust, London, UK; 3 Inflammatory Bowel Disease Unit, University of Calgary, Calgary, AB Canada; 4 Eli Lilly and Company, Indianapolis, IN USA; 5 Translational Gastroenterology Unit, Oxford University Hospitals NHS Foundation Trust and NIHR Biomedical Research Centre, Oxford, UK

**Keywords:** Bowel urgency, NRS, Patient-reported outcome, PRO, UC, Urgency

## Abstract

**Background:**

Bowel urgency, the sudden or immediate need to have a bowel movement, is a common, bothersome and disruptive symptom of ulcerative colitis (UC). UC treatment goals include control of urgency; however, it is not consistently assessed in UC clinical trials. The Urgency Numeric Rating Scale (NRS) is a new patient-reported measure to assess severity of bowel urgency in adults with UC developed in accordance with Food and Drug Administration guidelines.

**Methods:**

Qualitative interviews were used to develop Urgency NRS. The scale asks patients to report the immediacy status of their UC symptom over the past 24 h on an 11-point horizontal numeric rating scale [0 (No urgency) to 10 (Worst possible urgency)]. Higher scores indicate worse urgency severity. A 2-week diary study assessed floor and ceiling effects, test–retest reliability (intraclass correlation coefficient (ICC) (2,1) between Week 1 and 2), and construct validity (Spearman correlation using Week 1 scores). Weekly scores were calculated as mean score over each 7-day period.

**Results:**

Qualitative interviews with 16 UC patients (mean age 37.9 ± 11.6 years; 50% female; 56% White) confirmed relevance, content, and comprehensiveness. The 2-week diary study included 41 UC patients (mean age 44.2 ± 14.6 years; 51% female; 56% White). No ceiling or floor effects were identified. Test–retest reliability was high (ICC = 0.877). Average Urgency NRS and patient global rating of severity scores were highly correlated, with a moderate correlation between average Urgency NRS and stool frequency, demonstrating construct validity.

**Conclusions:**

Bowel urgency is a distinct symptom of UC. The Urgency NRS is a well-defined, content-valid, and reliable measurement of bowel urgency in adults with UC.

**Supplementary Information:**

The online version contains supplementary material available at 10.1186/s41687-022-00439-w.

## Background

Clinical manifestations of active ulcerative colitis (UC) include bloody diarrhea (with or without mucus), bowel urgency, tenesmus, abdominal pain, weight loss, fever, and malaise [1–4]. Bowel urgency, the sudden or immediate need to have a bowel movement, is one of the most common, bothersome, and disruptive symptoms experienced by patients with UC [5–15]. Bowel urgency is a key symptom for triggering clinical consideration of UC diagnosis and defining severity of disease activity in clinical practice [4, 16]. Cross-sectional studies report > 80% of patients with UC experience bowel urgency [13–15, 17], with up to half reporting having bowel urgency at least once a day [13, 14]. While the underlying mechanism(s) resulting in bowel urgency in UC is unclear, it has been associated with chronic inflammation, which causes changes in smooth muscle tone, sensitization of intramural and increased contractile responses in the rectum, as well as the development of submucosal fibrosis [18–20]. Consequently, bowel urgency may be seen in patients with UC regardless of the presence or absence of active disease [21, 22].

While UC can occur at any age, it often affects younger individuals during their most productive years [23]. The symptom burden associated with UC has a substantial negative impact on quality of life resulting in short- and long-term economic burden incurred from direct and indirect medical and work-associated costs [24–26]. Specifically, bowel urgency has been attributed to detriments in everyday functioning in the majority of patients with UC, and significantly impaired health-related quality of life [27]. The symptom has been related to limited participation in physical activity and exercise [28], decreased capacity to travel to and from work [29], and induced feelings of rising panic, distress, and embarrassment [30].

Recent updates to the American College of Gastroenterology (ACG) clinical guidelines [4] underscore control of bowel urgency as a component in defining treatment goals of remission in UC. Remission encompasses relief from symptoms (restoration of normal bowel frequency and control of bleeding and urgency) and resolution of intestinal inflammation as determined by endoscopy [31]. Both the ACG Activity Index and Simple Clinical Colitis Activity Index [32] include a measure of bowel urgency and are recommended for assessing disease activity severity in clinical practice [4].

Although patient [5–15] and professional clinical guidelines [4] identify bowel urgency as an important disease-related symptom signifying disease activity, the assessment of bowel urgency is not currently a recommended measured end point in clinical trials for inflammatory bowel disease (IBD) [2], therefore, it is not consistently assessed in UC clinical trials.

In line with principles of the Food and Drug Administration (FDA) Patient Focused Drug Development initiative [33], measurement of bowel urgency in clinical trials may better characterize the effect of drug products and describe the patient experience of one of the most meaningful and clinically relevant symptoms of UC, providing additional information beyond standard disease measures with respect to disease activity severity. Several questionnaires such as the Symptoms and Impacts Questionnaire for UC (SIQ-UC) [17] and Ulcerative Colitis Patient-Reported Outcomes Signs and Symptoms (UC-Pro/SS) diary [34] have recently been developed in accordance with FDA guidelines (2009) [35] for patient-reported outcomes (PROs). While each include an item consistent with the symptom of bowel urgency, these scales have multiple items and measure symptoms assessed by traditionally used scales, such as the Mayo Clinic Score [36], used as primary endpoints in UC clinical trials [2].

Numeric rating scales (NRSs) capture overall ratings of patients’ perceptions of subjective symptoms and may be especially useful when specific symptom experiences differ across individuals [37]. NRSs have been used in gastroenterology for assessment of abdominal pain in irritable bowel syndrome and analgesia during colonoscopy but are less commonly studied in IBD [38]. Similar to pain and analgesia, bowel urgency is also subjective and perceptions of the experience of urgency may vary across individual patients. Thus, an NRS is well suited for the assessment of urgency in UC.

The Urgency NRS is a new single-item, PRO measure to assess severity of bowel urgency in adults with UC and was developed in accordance with FDA PRO guidelines (2009) [35]. Findings from a literature review, interviews with gastroenterologists, and qualitative interviews with patients with UC confirmed that the symptoms of bowel urgency which were most salient to measuring UC disease activity [15] were used to generate the Urgency NRS. Here, we provide further details of the patient experience with bowel urgency and describe content validity confirmation and evaluation of reliability and construct validity of Urgency NRS. A Supplementary material movie file shows this in more detail (see Additional file 1).

## Methods

Development of a well-defined and reliable PRO measure is an iterative process [35], wherein evidence from patient research using qualitative methods (i.e. concept elicitation) to capture descriptions of disease experience (i.e. symptoms and impacts) are used [33]. Analysis of qualitative data examines data saturation, or when no emergent concepts are identified, providing confidence in experience importance and allowing for small sample sizes [33]. Once a PRO is generated based on patient input, patient confirmation (i.e. cognitive interviews) of the measure’s relevance to the disease and patient experience, and comprehensiveness is necessary to demonstrate content validity, thus providing credibility of its usefulness. Evidence to support the performance of a PRO measure is often characterized using quantitative research methods to evaluate measurement properties such as reliability and validity.

### Qualitative research (concept elicitation and cognitive interviews)

Qualitative research in adults with UC was conducted using concept elicitation and cognitive debriefing methods. Patient interviews were conducted to provide a comprehensive understanding of the clinically relevant symptoms and related impacts for patients with UC. Findings from a targeted literature review and expert input were used to inform the development of the semi-structured interview guide, which elicited information from patients about their lived experiences with UC [15]. Here we report further descriptions of bowel urgency from the patient perspective. Subsequent to concept elicitation, Urgency NRS was developed, and cognitive debriefing interviews were conducted in adults with UC to confirm content and comprehensiveness.

**Fig. 1:**
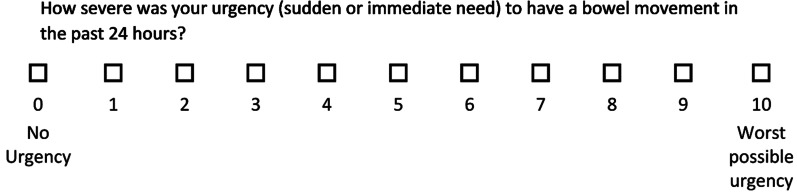
Urgency NRS, a patient-reported outcome measure to assess bowel urgency in adults with ulcerative colitis. For permission to reproduce or use Urgency NRS, please contact copyright@lilly.com

Urgency NRS (Fig. 1) is a new PRO measure for adult patients with UC to assess self-reported severity of bowel urgency. This purpose-designed PRO measure was constructed as a single item with both content coverage and simplicity in mind to maximize the potential for consistency and compliance. Urgency NRS was designed to assess changes in the severity of bowel urgency (sudden or immediate need). Severity of bowel urgency is defined by the patient’s perception of overall experience in which respondents consider the immediacy of bowel movement urgency severity over 24 h on an 11-point horizontal NRS ranging from 0 (‘no urgency’) to 10 (‘worst possible urgency’).

### Quantitative research (2-week diary study)

A real world 2-week diary study was conducted to collect ‘naturalistic’ data using Urgency NRS, to allow early quantitative validation and statistical interpretation. Patients participating in the diary study may also have participated in the qualitative concept elicitation or the qualitative cognitive debriefing research. The patient diary was administered electronically every day for a 14-day period. The core diary consisted of several, single-question items, including Urgency NRS, which patients were required to answer each day about their UC symptoms.

Additionally, on Day 1, patients were presented with an item requesting their consent to participate in the study. On Day 14, patients were presented with a 7-point Likert scale assessing their perceived change in symptoms over the 14-day period with responses ranging from ‘very much better’ to ‘very much worse’. Clinician-reported disease activity was also collected, wherein clinicians were asked to assess the severity of the patient’s UC on a scale of 1–5, where a score of 1 indicates ‘remission’ (no symptoms) and a score of 5 indicates ‘very severe’.

### Patient recruitment

Patients were recruited for the qualitative and quantitative research by clinical staff participating in a third-party recruitment agency network within the continental United States. Clinical sites were in St Louis, Chicago, New Orleans, and Baltimore. All sites received study-specific training prior to the study launch. Target recruitment quotas were used to guide recruitment to ensure that the study sample was adequately representative of the patient population.

Key inclusion criteria included 18 years or older and UC of at least 3 months duration confirmed by sigmoidoscopy or colonoscopy. Patients with previous diagnosis of Crohn’s disease, indeterminate colitis, radiation colitis, or diverticular-associated colitis that could interfere with the participant’s ability to evaluate the symptoms and impacts of their UC and surgical resection were excluded.

### Analytic approach

All qualitative interviews were conducted in person in a one-on-one setting by trained interviewers using a semi-structured discussion guide, digitally recorded, and transcribed verbatim for the purposes of analysis. A systematic content analysis approach was used to analyze the qualitative data from interviews using ATLAS.ti v7.0 (ATLAS.ti Scientific Software Development GmbH) for coding. To ensure that the concepts elicited from patients had been fully explored during the interviews, concept saturation was assessed to ensure no emergent concepts were identified and are described elsewhere [15]. A full coding list and saturation table was produced and reviewed by the research team and disease area experts, to ensure agreement that saturation had been met for all key concepts.

Quantitative data analyses were completed using SAS (SAS Institute; Cary, NC, version 9.4). Weekly average scores were calculated for Urgency NRS as the mean item score over each 7-day period (Week 1: Day 1–7; Week 2: Day 8–14). Weekly average scores were calculated for patients with at least 4 of 7 days of complete diary data. There was no imputation for missing data, but patterns of missing data and compliance were explored.

Descriptive analyses of demographic and health information data were conducted using Day 1 data. Frequency, mean, standard deviation (SD), median, minimum, maximum, and number of missing data were calculated for continuous variables. For categorical variables, the number and percentage of patients endorsing each category were reported.

Frequency and percentage of endorsements were presented for each response option on Urgency NRS for Day 1 to Day 14. Floor and ceiling effects were displayed as the proportion of patients who responded at the lowest (0 = ‘no urgency’) and highest response option (10 = ‘worst possible urgency’), respectively.

Test–retest reliability was assessed using the Shrout and Fleiss intraclass correlation coefficient (ICC) (2,1) where a test–retest reliability of ≥ 0.75 or ≥ 0.80 has been suggested as evidence of substantial agreement for a scale to be used in detecting group mean differences [39, 40]. To assess the weekly stability of Urgency NRS, the ICC between Week 1 and Week 2 average scores was calculated. Bootstrapping (sampling with replacement) was used to create multiple datasets (n = 1000) each containing a sample size equivalent to the initial sample to estimate the ICC and associated 95% confidence intervals (CIs). A sensitivity analysis for test–retest reliability compared weekly scores for patients reporting ‘no change’ for their assessment of perceived change in symptoms over the 14-day period.

Convergent and discriminant validity were assessed by Spearman rank-based correlation coefficients at the end of Week 1 (average of 7 days of diary data) between Urgency NRS scores and stool frequency and Patient Global Rating of Severity (PGR-S) scores based on the hypothesis that there would be moderate to large correlations. Cohen’s conventions (any correlation > 0.5 is large, 0.3 to ≤ 0.5 is moderate, 0.1 to < 0.3 is small, and < 0.1 is insubstantial) were used to interpret the results [41]. Stool frequency was measured using an electronic daily diary to record bowel movement count in the past 24 h. Stool was defined as a bowel movement, the passing of blood alone, blood and mucus, or mucus only; PGR-S (a 1-item questionnaire to assess the patients’ rating of their disease symptom severity over the past 24 h on a 6-point scale, where a score of 1 indicates no symptoms (that is, “none”) and a score of 6 indicates that the patient’s symptom(s) are “very severe”) was using an electronic daily diary.

### Ethical considerations

Before recruitment of subjects, all study documents were submitted and approved by Copernicus Group Independent Review Board®, approval number Clinical Outcomes Solutions (COS)1-17-209. Interviews were conducted after the participant had signed and returned the informed consent document to COS.

## Results

Details presented from concept elicitation interviews further expand on patient experiences of bowel urgency as a symptom of UC described previously [15]. Evidence gathered from these interviews were the basis for development of Urgency NRS. Cognitive debriefing interviews were conducted to confirm content and comprehensiveness of Urgency NRS to establish content validity. Quantitative evaluation provided evidence to support adequate reliability and construct validity.

### Qualitative research: concept elicitation interviews

In total, 21 adults participated in the concept elicitation interviews. Patients had a mean age of 45 ± 15.1 years, the majority were female (52%), 67% reported their race as White, and 91% reported their ethnicity as non-Hispanic/Latino (Table 1). The mean duration since diagnosis was 9.3 years. Clinician-reported disease severity ranged from mild to severe, with 48% of patients reporting having experienced a UC flare the past 2 weeks.

**Table 1:** Patient demographics and clinical characteristics

Variables	Concept elicitation (N = 21)	Cognitive interviews (N = 16)	2-week diary study (N = 41)
Age			
Mean (SD)	44.9 (15.10)	37.9 (11.6)	44.2 (14.64)
Gender, n (%)			
Male	10 (47.6)	8 (50.0)	20 (48.8)
Female	11 (52.4)	8 (50.0)	21 (51.2)
Race, n (%)			
White	14 (66.7)	9 (56.2)	23 (56.1)
Black/African American	6 (28.6)	5 (31.3)	14 (34.2)
Other	1 (4.7)	2 (12.5)	4 (9.7)
Ethnicity, n (%)			
Hispanic/Latino	2 (9.5)	3 (18.8)	4 (9.8)
Not Hispanic/Latino	19 (90.5)	13 (81.2)	37 (90.2)
Education, n (%)			
Did not complete high school	0	1 (6.2)	1 (2.4)
High school diploma	5 (23.8)	1 (6.2)	8 (19.5)
Some college or certification program	4 (19.1)	5 (31.3)	10 (24.4)
College degree	7 (33.3)	4 (25.0)	14 (34.2)
Graduate degree	5 (23.8)	4 (25.0)	6 (14.6)
Other	0	1 (6.3)	2 (4.9)
Employment, n (%)			
Employed full-time (≥ 40 h per week)	15 (71.4)	11 (68.8)	28 (68.3)
Employed part-time (< 40 h per week)	2 (9.5)	4 (25.0)	4 (9.8)
Homemaker	1 (4.7)	0	1 (2.4)
Retired	2 (9.5)	1 (6.2)	6 (14.6)
Unemployed	1 (4.7)	0	1 (2.4)
Other	0	0	1 (2.4)
Years since UC diagnosis			
Mean (SD)	9.3 (16.10)	5.3 (5.95)	6.0 (8.16)
Median	4.1	3.6	4.1
Min/Max	0.3, 64.1	0.3, 24.6	0.3, 45.6
Hospitalization in past 6 months, n (%)	3 (14.3)	2 (12.5)	3 (7.3)
Clinician reported UC severity, n (%)			
Mild	5 (23.8)	3 (18.8)	12 (29.3)
Moderate	8 (38.1)	3 (18.8)	10 (24.4)
Severe	8 (38.1)	10 (87.5)	19 (46.3)
UC flare in past 2 weeks, n (%) (patient-reported)	10 (47.6)	5 (31.3)	12 (29.3)

*N/A* not applicable, *N* total sample size, *n* sample size from the total group, *SD* standard deviation

### Patient description of bowel urgency

Having a sudden or immediate need to have a bowel movement was discussed spontaneously by 19/21 patients. The need to go to the bathroom suddenly was described as distinct from, but often experienced concurrently with, a high frequency of bowel movements. Patients indicated that not only were bowel movements often frequent, but they also involved having to use the bathroom quickly. The sudden or immediate need for a bowel movement was reported as being both an ‘acute’ and a ‘chronic’ symptom of UC.

Patients discussed the need to plan around their UC in relation to sudden or immediate bowel movements, specifically having knowledge of local bathrooms when a UC attack occurs. The immediacy with which bowel movements could occur was essential information to avoid accidents, or not being able to make it to a toilet in time. It was described by 5 patients as having a negative effect on their work life, as patients would have to use the bathroom suddenly and thus needed to be near a bathroom. Bowel urgency was described as causing patients to have to stop what they were doing and find a bathroom immediately, thus requiring advance knowledge of toilet locations when out of the house.

### Patient descriptions of impacts of bowel urgency

Impact on social functioning was described spontaneously by 18/21 patients as having to plan around UC. Patients described this concept as being closely related to the impact of UC on their social lives and relationships with others. As a result, patients would have to plan for bathroom breaks due to the urgency and frequency of bowel movements, which often either limited what they could do or meant that that could not participate in the same activities as others.

Worry, anxiety, and fear due to UC were discussed by 17/21 patients as being impacts on their emotional well-being. Specifically, patients mentioned worrying about having a sudden or immediate need to have a bowel movement, having to plan and schedule around their symptoms, and the unpredictability of when their next flare might occur. Managing this and being preoccupied by their condition in this way was described as leading patients to feel worried, anxious, and afraid about when symptoms would occur.

Patients (13/21) discussed that sleep disturbances occurred mainly during a UC attack when UC symptoms were at their worst, but that they could also occur between UC attacks or ‘flares.’ When discussing difficulties staying asleep, 5 patients commented that the main reason for this was having to use the bathroom frequently and suddenly during the night, with some reporting waking 3–5 times a night. As a result, patients experienced fewer hours of sleep, which impacted their ability to work the next day and perform other activities in general.

### Qualitative research: cognitive interviews

In total, 16 adult patients with UC participated in the concept debriefing interviews. Patients had a mean age of 37.9 ± 11.6 years, there was an even proportion of males and females (50%), the majority reported their race as White (56%), and 81% reported their ethnicity as non-Hispanic/Latino (Table 1). The mean duration since diagnosis was 5.3 years. Clinician-reported UC severity ranged from mild to severe with most patients (87%) having severe disease. Thirty-one percent of patients reported having experienced a UC flare the past 2 weeks.

During the cognitive debriefing interviews, patients with UC were asked to identify any words or phrases they had difficulty interpreting. The interviewers then guided patients through the electronic version of Urgency NRS asking them to paraphrase the meaning of the question. All patients (16/16) demonstrated that they understood this item and described experiences indicating that ‘urgency’ was a relevant concept for UC.

There were no issues raised in relation to the recall period and it was deemed relevant. The recall period of ‘past 24 h’ was considered appropriate, made sense, and was relevant to the symptom of bowel urgency. Patients were able to make ratings based strictly on this recall period and reported no difficulty in remembering their experiences over that period.

Additionally, there were no issues raised in relation to the response options and they were deemed relevant. No patients identified difficulty in making a rating of severity on the 0 to 10-point NRS. Patients used lower ratings of the scale to indicate less severity of bowel urgency based on their overall experience and cumulative impact of bowel movement urgency severity over 24 h. Likewise, higher ratings were used to describe greater severity of UC-related bowel urgency based on their overall experience and cumulative impact of bowel urgency severity over 24 h.

As part of the cognitive debriefing interviews, qualitative feedback was gathered from patients in relation to the minimal amount of change on the 0 to 10-point NRS that they would consider ‘meaningful’. Just over half (9/16) commented that they would consider a 1-point score change on Urgency NRS meaningful, while a third (3/16) felt that a 2-point score change was meaningful. Most patients suggested that a 1- or 2-point reduction would be better because their symptoms would improve, and any improvement in the feelings of bowel movement urgency would be better than what they have now.

### Quantitative 2-week diary study

In total, 41 adult patients with UC participated in the 2-week daily diary pilot study. Patients had a mean age of 44.2 ± 14.6 years, there were slightly more female patients (51%), the majority reported their race as White (56%), and 90% reported ethnicity as non-Hispanic/Latino (Table 1). The mean duration since diagnosis was 6.0 years. Clinician-reported UC severity ranged from mild to severe with many patients reporting severe UC (46%), while 29% patients reported having experienced a UC flare the past 2 weeks.

**Fig. 2:**
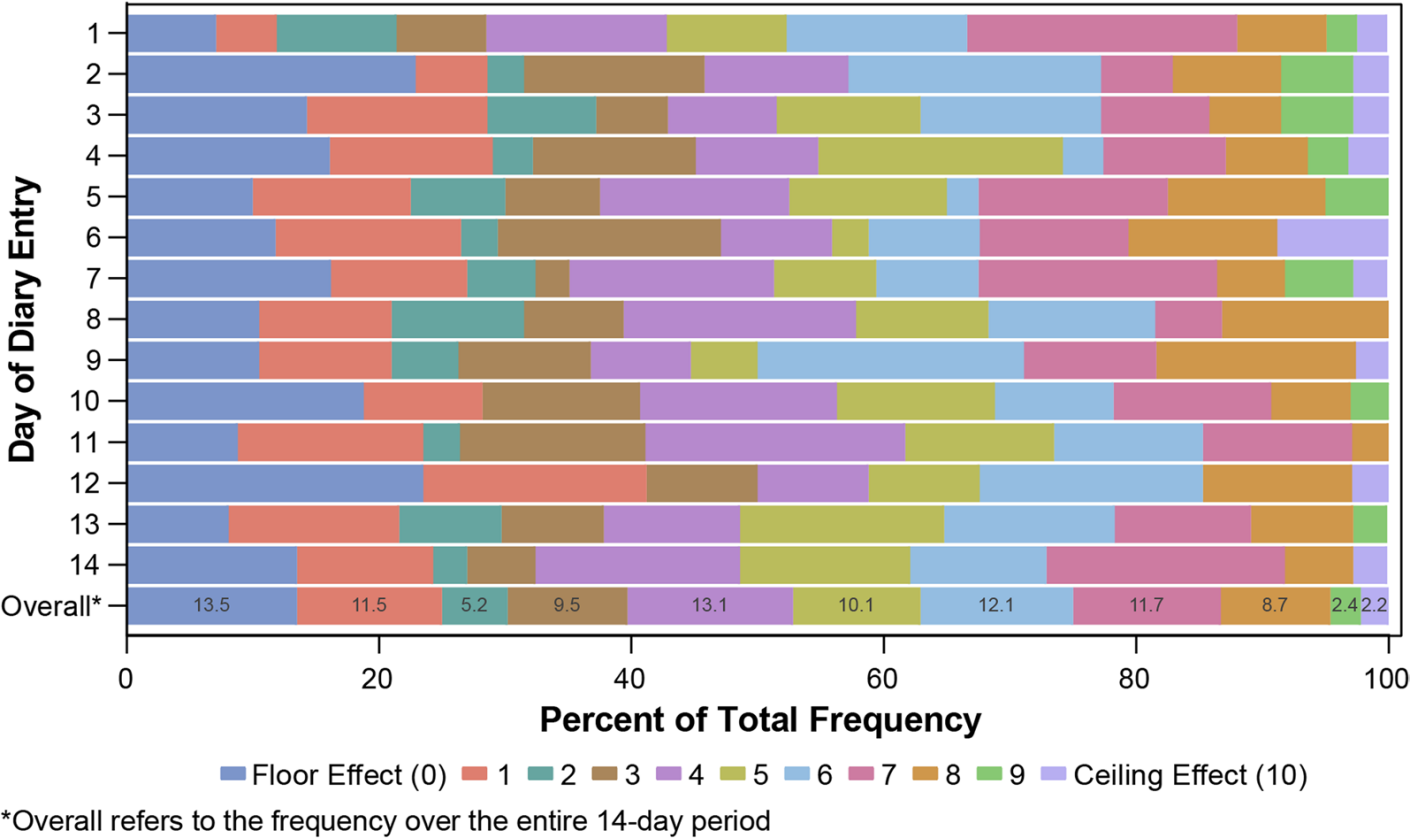
Item distribution and floor/ceiling effects for Urgency NRS scores by day of 2-week diary study

All patients met the compliance threshold of having completed at least 4 of 7 days in Week 1 and were included in weekly average computations. In Week 2, 1 patient completed only 3 days and was thus removed from all weekly calculations for that week. Most (30/41) patients completed 11 or more diary days. Average Urgency NRS scores reported for the sample were 4.47 and 4.20 for Week 1 and Week 2, respectively (Table 2). Daily scores ranged from 0 to 10, with medians of 5, and most responses were between 0 to 7 for the overall sample, tapering off on responses 8, 9, and 10 (Fig. 2). No ceiling or floor effects were observed.

**Table 2:** 2-Week diary study average Urgency NRS scores

*Day 1*	
N	41
Mean (SD)	4.90 (2.60)
Median	5
Min–Max	0–10
*Day 14*	
N	37
Mean (SD)	4.32 (2.74)
Median	5
Min–Max	0–10
*Week 1 average*	
N	41
Mean (SD)	4.47 (2.46)
Median	4.60
Min–Max	0–8.33
*Week 2 average*	
N	40
Mean (SD)	4.20 (2.27)
Median	4.45
Min–Max	0–7.86

*N* total sample size, *NRS* numeric rating scale, *SD* standard deviation

Test–retest reliability of Urgency NRS using the bootstrapped samples was high (ICC = 0.877), with a 95% CI of 0.770 to 0.947 for the bootstrapped estimate (Table 3). A total of 25 patients reported ‘no change’ in their perceived symptoms over the 14-day period and formed the sample for the sensitivity analysis. Test–retest reliability was similarly high within this sample (ICC = 0.867, 95% CI: [0.583, 0.968]).

**Table 3:** 2-week diary study, Urgency NRS test–retest reliability

*Test–retest (week 1–week 2)*	
N	41
ICC^†^	0.877
95% CI	(0.770, 0.947)
*Test–retest (sensitivity)*	
N	25
ICC^†^	0.867
95% CI	(0.583, 0.968)

*CI* confidence interval, *ICC* intraclass correlation coefficient, *N* total sample size, *NRS* numeric rating scale

*ICC and 95% CI were estimated through bootstrap (n = 1000)

^†^ICC was calculated using the ICC (2,1) model of Shrout and Fleiss (1979)

High correlations (r = 0.8) were observed between average Urgency NRS scores and average PGR-S scores in Week 1 (Table 4), suggesting bowel urgency is top of mind when patients consider their overall symptom severity. A moderate correlation (r = 0.5) was observed between average Urgency NRS score and average number of stools (stool frequency) in Week 1 (Table 4), indicative of bowel urgency being a distinct symptom from stool frequency.

**Table 4:** Correlation between average Urgency NRS and other outcome assessments scores in week one

Outcome assessment	Sample size	Spearman correlation (95% CI)
Average PGR-S score	41	0.802 (0.651, 0.888)
Average number of daily stools	41	0.516 (0.241, 0.707)

*CI* confidence interval, *NRS* numeric rating scale, *PGR-S* patient global rating of severity

## Discussion

Development of a well-defined and reliable PRO measure demands evidence directly from patient research using qualitative methods to capture descriptions of disease experience in their own words [33]. Once a PRO is generated based on patient input, evidence must be provided for patient confirmation of the relevance to the disease and patient experience, and comprehensiveness. Once content validity has been established, performance metrics such as reliability and validity can then be characterized. Urgency NRS is a newly developed single-item, PRO measure to assess severity of bowel urgency in adults with UC. The basis for development incorporated these principles by integrating findings from a literature review, interviews with gastroenterologists, and qualitative concept elicitation interviews to solicit patient experiences with patients with UC that confirmed bowel urgency symptom severity most pertinent to measuring UC disease activity [15].

Ninety percent of patients participating in concept elicitation interviews spontaneously expressed bowel urgency to be a common, bothersome, and disruptive symptom of UC. The sudden or immediate need to have a bowel movement was described as a distinct symptom, but often experienced concurrently with increased stool frequency. Patients described significant negative impacts of bowel urgency. Socially, patients are limited by bowel urgency and stool frequency in what activities they could participate. Emotionally, having to manage bowel urgency and knowing the location of the nearest bathroom lead patients to feel worried, anxious, and afraid about when symptoms would occur. Patients experience fewer hours of sleep from waking at night from the need to immediately have a bowel movement, which subsequently impacts their ability to work the next day and perform other activities in general.

The semi-structured interviews in this study allowed patients with UC to describe bowel urgency in their own words. Patient descriptions of a score of 0 on Urgency NRS included “fine” and feeling the need to go but not having to act on it. Patient descriptions of a score of 10 also varied and included the need to stay in the bathroom, getting to the bathroom as quickly as possible, not being able to make it to the bathroom, and having an accident. The Urgency NRS, which includes anchors at each end of “no urgency” (score of 0) and “worst possible urgency” (score of 10), was found to be easily understood, confirmed to include appropriate response options, and doesn’t rely on specific descriptors that may not be relevant to all patients due to the varied personal experiences of urgency.

During cognitive debriefing interviews, all patients felt Urgency NRS was easy to understand with a suitable recall period and that the response options were appropriate. Patients did not report any problems interpreting or using Urgency NRS; thus, no changes were deemed necessary. Overall, the cognitive interviews revealed that the item adequately captured the concept as intended and is, overall, easily understood and appropriate from a patient perspective. When asked to describe the level of meaningful change on the 0 to 10-point NRS, most patients consistently selected a 1- to 2-point change as the minimum amount of change they would want to experience. It was acknowledged that a bigger change would be ideal, but most patients would be satisfied with any improvement of the symptom.

Completion compliance rates observed in the quantitative 2-week diary study were high for all patients, suggesting ease of use for Urgency NRS. No ceiling or floor effects were observed as response distributions were reasonably uniform during the 14 days. The evaluation of test–retest reliability properties through bootstrap revealed high ICC, with sensitivity analyses among patients reporting ‘no change’ for their assessment of perceived change in symptoms over the 14-day period showing similar stability. High to moderate correlations with PGR-S scores and stool frequency demonstrate adequate construct validity, suggesting bowel urgency is top of mind when patients consider their overall disease severity, and is a distinct symptom from stool frequency.

Although the burden and impact of bowel urgency as a symptom of UC have been described in multiple publications, patients with UC may be hesitant to report symptoms such as urgency due to their embarrassing nature and physicians may not ask about all symptoms [14]. ACG recently updated their clinical guidelines suggesting the inclusion of a measure of bowel urgency when assessing severity of disease activity in clinical practice, citing use of the ACG Activity Index or the Simple Clinical Colitis Activity Index [4]. Since Urgency NRS is a quick and validated scale, it may be a useful tool for the assessment of urgency in clinical practice in addition to its utility in clinical trials.

Currently, the assessment of bowel urgency is not a recommended end point in UC clinical trials [2], therefore, it is not consistently assessed. Traditional symptom endpoints such as stool frequency and rectal bleeding have been included in the evaluation of disease activity, with improvement of these symptoms as an indication of treatment benefit of new drug products. The development of newer PROs such as the SIQ-UC [17], UC-Pro/SS [34] and Ulcerative Colitis Symptoms Questionnaire [42] for use in clinical trials provides a means for assessing bowel urgency, however, with the added burden of redundant symptom measurement of stool frequency and rectal bleeding.

In line with principles of the FDA Patient Focused Drug Development initiative [33] which call for the incorporation of patient experience into drug development, Urgency NRS is a simple, single-item questionnaire that directly asks patients to rate the severity of their urgency (sudden or immediate need) to have a bowel movement over the past 24 h. The use of Urgency NRS within a UC clinical trial utilizing a daily diary can account for the nature of UC relapsing–remitting course and negates the need to assess symptom frequency. The short recall period reduces recall bias, and the use of an NRS provides a finer gradation of response options and can be readily applied cross-culturally and across languages. Measurement of bowel urgency, one of the most meaningful and clinically relevant symptoms of UC, in clinical trials will characterize the effect of drug products, consistent with FDA guidance to relieve signs and symptoms of active disease [2]. Urgency NRS is a well-defined and content-valid PRO measure intended for use in clinical trials to assess meaningful treatment benefit in UC.

There were several limitations of our research. Most patients were White and non-Hispanic/Latino which could limit generalizability to patients with UC. Additionally, representativeness of the study population is limited in that only a few patients had less than a high school education and the majority were employed full-time. Approximately only one-third of the population experienced a UC flare within the past 2 weeks prior to study participation which could introduce recall bias to descriptions of UC symptoms and impacts. However, this seems unlikely given that 90% of patients participating in the concept elicitation interviews spontaneously described bowel urgency and related impacts. Our research did not evaluate Urgency NRS responsiveness to change nor scores constituting clinically meaningful within-patient improvement or responder definitions; additional research is currently ongoing to characterize these properties.

## Conclusion

Patients endorse bowel urgency as a common, bothersome, and disruptive symptom of UC, distinct from traditionally measured symptoms of stool frequency and rectal bleeding. Bowel urgency is associated with significant negative impacts on social and emotional well-being. Urgency NRS is a PRO measure developed in accordance with FDA PRO guidelines [35]. It is a well-defined, content-valid scale for adults with UC that captures the spectrum of bowel urgency severity. Inclusion of Urgency NRS in clinical trials may better characterize the effect of drug products and describe the patient experience of one of the most meaningful and clinically relevant UC symptoms, providing additional information beyond standard disease measures with respect to disease activity severity. The simplicity of Urgency NRS may also allow for a means to incorporate assessment and discussion of bowel urgency in routine clinical practice to help clinicians assess and manage UC disease activity in accordance with clinical guidelines.

## Supplementary Information

**Additional file 1.** Video abstract: Development of the Urgency Numeric Rating Scale to Assess Bowel Urgency presented by Dr. Marla C. Dubinsky.

## Abbreviations

ACG: American College of Gastroenterology
CI: Confidence intervals
COS: Clinical outcomes solutions
FDA: Food and drug administration
ICC: Intraclass correlation coefficient
NRS: Numeric Rating Scale
PRO: Patient-reported outcomes
SD: Standard deviation
SIQ-UC: Symptoms and impacts questionnaire for UC
UC: Ulcerative colitis
UC-Pro/SS: Ulcerative colitis patient-reported outcomes signs and symptoms

## Declarations

### Availability of data and materials

The datasets generated and/or analyzed during the current study are not publicly available due to the sensitive nature of the questions asked in this study but are available from the corresponding author on reasonable request.
